# Potential of sphingosine-1-phosphate in preventing SARS-CoV-2 infection by stabilizing and protecting endothelial cells: Narrative review

**DOI:** 10.1097/MD.0000000000029164

**Published:** 2022-04-15

**Authors:** Rongzhi Zhang, Qiang Wang, Jianshe Yang

**Affiliations:** a Department of Anesthesiology, Lanzhou University Second Hospital, Lanzhou, Gansu, China; b Gansu Medical College, Pingliang, Gansu, China; c Shanghai Tenth People's Hospital, Tongji University School of Medicine, Shanghai, China.

**Keywords:** coronavirus disease 2019, endothelial cells, infection, severe acute respiratory syndrome coronavirus 2, sphingosine-1-phosphate

## Abstract

Coronavirus disease 2019, caused by severe acute respiratory syndrome coronavirus 2 (SARS-CoV-2), has spread worldwide, resulting in over 250 million infections and >5 million deaths. Most antiviral drugs and vaccines have shown limited efficacy against SARS-CoV-2. Clinical data revealed that except for the large number of self-healing mild cases, moderate and severe cases mostly survived after supportive treatment but not specific drug administration or vaccination. The endothelial system is the first physiological barrier, and its structural stability is of critical importance in conferring disease resistance. Membrane lipid components, particularly sphingosine-1-phosphate (S1P), play a central role in stabilizing the cell membrane.

Here, we used “Boolean Operators” such as AND, OR, and NOT, to search for relevant research articles in PubMed, then reviewed the potential of S1P in inhibiting SARS-CoV-2 infection by stabilizing the endothelial system, this is the major aim of this review work.

Reportedly, vasculitis and systemic inflammatory vascular diseases are caused by endothelial damage resulting from SARS-CoV-2 infection. S1P, S1P receptor (SIPR), and signaling were involved in the process of SARS-CoV-2 infection, and S1P potentially regulated the function of EC barrier, in turn, inhibited the SARS-CoV-2 to infect the endothelial cells, and ultimately has the promising therapeutic value to coronavirus disease 2019.

Taken together, we conclude that maintaining or administering a high level of S1P will preserve the integrity of the EC structure and function, in turn, lowering the risk of SARS-CoV-2 infection and reducing complications and mortality.

## 1 Introduction

**Figure 1:**
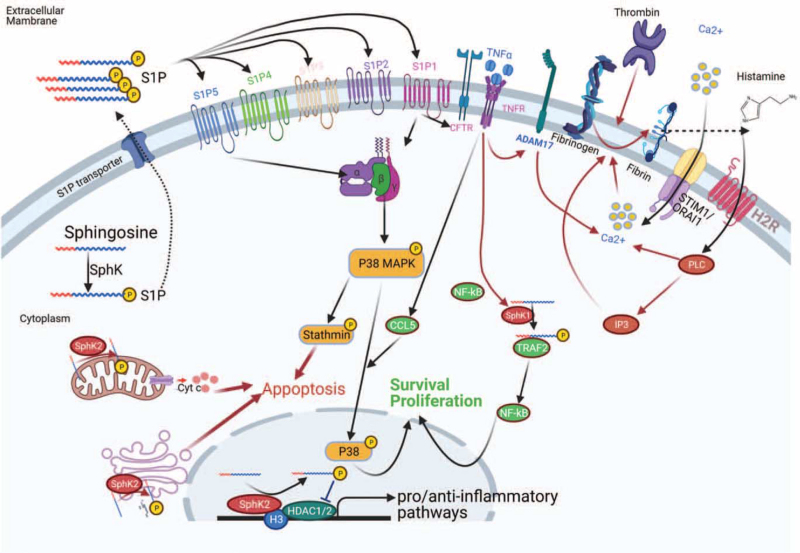
Sphingosine-1-phosphate pathway modulates endothelial cell apoptosis and proliferation via S1P1–S1P5, regulated by activated SphK during SARS-CoV-2 infection.

Lipids are important constituents of the cell membrane and include diverse multifunctional components that maintain flexibility and integrity of the membrane, enabling it to adapt to complex extracellular environments.^[1]^ Lipids are asymmetrically distributed across membrane lipid bilayer to execute essential biochemical functions. The predominant forms of lipids are the extracellular phospholipids phosphatidylcholine, sphingomyelin, intracellular phosphatidylserine, phosphatidylethanolamine, and phosphatidylinositol.^[2]^ Sphingosine-1-phosphate (S1P) is a derivative of sphingomyelin, which has 5 multifunctional subtypes modulated by sphingosine kinase (SphK, including SphK1 and SphK2), and regulates diverse biological functions, such as stimulating cell proliferation (SphK1) and promoting apoptosis (SphK2).^[3]^ Endothelial cells (ECs) act the first barrier and are eventually attacked foremost by severe acute respiratory syndrome coronavirus 2 (SARS-CoV-2). S1P, modulated by activated SphK1 and SphK2, may stimulate cell proliferation and promote apoptosis, thereby ameliorating the outcome of SARS-CoV-2 invasion (Fig. 1).

## 2 Methods

### 2.1 Literature research

In order to address this issue, we used “Boolean Operators” such as AND, OR, and NOT, to search for relevant research articles in PubMed for the chain of S1P→complete endothelial system→against SARS-CoV-2 invading, and then fetched and filtered relevant articles. Briefly, we searched for relevant research papers on the application of sphingosine-1-phosphate, in preventing SARS-CoV-2 infection through stabilizing and protecting ECs, as a treatment for coronavirus disease 2019 (COVID-19). A total of 667 articles, reviews, clinical trials, meta-analysis, and randomized controlled trials (all in English language) containing the keywords “sphingosine-1-phosphate OR endothelial AND SARS-CoV-2 OR COVID-19” were screened and selected for analysis. Three reviews/systematic reviews included all the 3 keywords, while 15 included “COVID-19/SARS-CoV-2, sphingosine-1-phosphate,” and 640 contained “endothelial, COVID-19/SARS-CoV-2.” Thirty-two books and documents, clinical trials, meta-analyses, and randomized controlled trials contained all the 3 keywords. These results imply that studies involving sphingosine-1-phosphate and COVID-19/SARS-CoV-2 are scanty, and further studies should be conducted on a larger scale.

Concerning about the ethical issues, we declared that this work does not require ethical approval because it is a literature review of human and animal experimental and clinical ethics.

## 3 Results

### 3.1 S1P, S1P receptor (SIPR), and signaling

S1P, derived from cell membrane sphingolipids, is enriched in the circulatory system, with wide biological functions, including the regulation of embryonic and postnatal organ development and inflammatory diseases by combining with G protein-coupled S1PRs (including 5 subtypes from S1PR1 to S1PR5). S1PRs regulate S1P-associated physiological processes, and S1PR signaling can drive the occurrence of several diseases (Fig. 1). Studies on lipid mediators and S1PR-targeted drugs reveal fundamental principles of complex S1P actions and guide the progress of therapeutic directions in multiple systematic diseases.

With the development of evolutionary theory, metabolites of membrane lipids are not merely regarded as common nonfunctional terminal substances; they can act as extracellular ligands for G protein-coupled receptors (GPCRs), which modulate numerous intracellular signaling pathways. This integration indicates a communication bridge between membrane phospholipid derivatives and intercellular responses.^[4]^ The platelet-activating factor (a bioactive substance synthesized from membrane phospholipids) can activate GPCRs to initiate an allergic response.^[5]^ Moreover, S1Ps, secreted by ECs, act on specific GPCRs in nearly all cell populations.^[4]^

S1P was first discovered and identified as a terminal metabolite of sphingolipids.^[6]^ S1P has been speculated to play a role of a classical second messenger, such as diacylglycerol or calcium ion (Ca^2+^).^[7,8]^ The 5 receptors bind to extracellular S1Ps to regulate specific physiological and pathological processes.^[4]^ S1PR-associated intracellular signaling pathways are comodulated by the protein kinase AKT and guanosine triphosphatases, which regulate different cell behaviors.^[9]^ S1P maturation is a complex process that occurs across the bilayer cell membrane. To date, we have elucidated the extracellular part of signal transduction and material transmission; however, the intracellular part remains unclear. S1P binding regulates the activities of targeted molecules (most of them are specific S1P receptors), such as the cytosolic signal transduction mediator tumor necrosis factor alpha receptor-associated factor 2,^[10]^ chromatin modification-associated histone deacetylase-1,^[11]^ mitochondrial regulator prohibitin-2,^[12]^ atypical protein kinase-C,^[13]^ and telomere reverse transcriptase catalytic subunit.^[14]^ All these molecules and related bioprocesses are associated with cellular activities, including cell proliferation, apoptosis, and disease development. However, intracellular S1P signaling and relevant physiological events remain unclear because of the lack of genetic studies associated with intracellular S1P targets.^[15–18]^

S1P has multiple functions in developmental, physiological, and pathological aspects because of its unique physicochemical properties.^[19]^ It can modulate inflammation owing to its antioxidative potential, which makes it more capable of the influx of oxidative cells. S1P, being a lipid, is difficult to be dissolved in the aqueous phase. A conjugation with proteins enhances its solubility and enriches its biological functions, eventually making it feasible to get transported through circulation and combine with S1PRs.^[20]^ Furthermore, these conjugations promote the formation of S1P gradients (S1P–S1P5), which are located in different cellular apartments, and can activate specific S1PR receptors.^[21–23]^ Diverse S1P functions have been precisely illustrated through in vivo or in vitro studies in humans and animals, with congenital conditions related to S1PRs, transporters, and S1P metabolic enzymes either dysfunctional or mutated.^[4,24,25]^

S1P-regulated processes are widely associated with a vast spectrum of disorders, including cardiovascular and autoimmune diseases, inflammation, cancer, and fibrosis.^[4]^ Fingolimod, an S1PR-targeted drug approved by the US FDA, has been applied in treating multiple sclerosis, because of its ability to promote cell apoptosis and specifically inhibit hyperplasia.^[26]^ Recently, fingolimod administration in patients with severe COVID-19 infection alone or concurrent with MS^[27–30]^ has shown positive efficacy in disease control, while the symptoms were exacerbated with withdrawal of fingolimod administration.^[31]^ In some clinical trials, S1P/S1PR signaling has been found to play active roles in the nervous system and heart function.^[32,33]^

### 3.2 S1P in regulating the function of EC barrier

Endothelial barrier function is strictly regulated by receptors on the plasma membrane through binding or removal of specific ligands to open or close specific material transport channels. Its dysfunction may cause several diseases because of the abnormal influx or outflow of materials, such as Ca^2+^, which are crucial during SARS-CoV-2 attack and its cellular entry facilitated by transmembrane protease serine 2 (TMPRSS2) membrane receptor,^[34]^ while Ca^2+^ is tightly associated with TMPRSS2.

Receptor-regulated endothelial barrier function is influenced by ubiquitous activation of Ca^2+^ signaling, which subsequently regulates phospholipase C-coupled receptor ligation. This model has been verified in experiments with smooth muscles, by initiating endothelial contraction and generating inter-endothelial gaps.^[35]^ A potential mechanism of Ca^2+^-induced endothelial contraction has been adopted to elucidate GPCR agonist-mediated regulation of EC barrier function.^[36]^

Thrombin and histamine are endogenous GPCR agonists that affect endothelial barrier function,^[37]^ and this outcome can be reversed by S1P.

The cystic fibrosis transmembrane conductance regulator affects the stability of the lung endothelial barrier. Conversely, cystic fibrosis transmembrane conductance regulator dysfunction may aggravate lung inflammation by enhancing EC permeability. However, this negative effect is ameliorated by S1P treatment. S1P and S1PRs are expressed ubiquitously in tissues and contribute to pro- and anti-inflammatory effects of S1P signaling modulation.^[38]^ For example, SphK1 is secreted and enriched in the cytosol, plasma membrane, and extracellular matrix, while SphK2 acts within the cells during inflammation.^[39,40]^ SphK1–S1P signaling is tightly associated with autoimmune diseases, such as rheumatoid arthritis and inflammatory bowel disease, and targets proinflammatory cytokines.^[41]^ TNF-mediated SphK1 induces S1P secretion; thereafter, S1P binds to TNF receptor associated factor 2 to induce K63 polyubiquitylation of receptor-interacting protein to activate nuclear factor kappa B (NF-κB).^[10]^ The SphK1–S1P axis modulates TNF-mediated C-C Motif Chemokine Ligand 5 through mitogen-activated protein kinase pathway without the involvement of NF-κB signaling.^[42]^ SphK1 and SphK2 are not essential for TNF-mediated NF-κB activation in macrophages (Fig. 1).^[18]^

### 3.3 ECs and SARS-CoV-2

SARS-CoV-2 infection exacerbates lesions in ECs. The pathophysiology of endothelial dysfunction increases awareness of COVID-19-associated mortality.^[43]^ A direct infection of engineered human blood vessel organoids has been observed.^[44]^ Varga et al^[45]^ have reported that ECs are involved in SARS-CoV-2 infection. They provided evidence of viral invasion into ECs and subsequent inflammation by recruitment of neutrophils and mononuclear cells to ECs. They also identified viral inclusions in ECs of a transplanted kidney, using electron microscopy; another severe patient who died from multi-organ failure showed lymphocytic endotheliitis in same organs. Histology of small intestine resection in a COVID-19 patient with mesenteric ischemia showed prominent endotheliitis of the submucosal vessels, a strong evidence of viral infection of ECs and mononuclear cell infiltration. COVID-19-induced endotheliitis systematically impaired microcirculation in COVID-19 patients.^[45]^ Severity of COVID-19 was highly correlated with emergent cytokine secretion and immune cell response based on EC activation.^[46]^ Since ECs play a vital role in maintaining vascular and blood homeostasis, their dysfunction may be involved in thrombo-inflammatory development, ultimately resulting in vasculopathy and acute respiratory distress syndrome.^[47]^

### 3.4 Process of SARS-CoV-2 entry into ECs

Angiotensin-converting enzyme 2 (ACE2) and TMPRSS2 facilitate SARS-CoV-2 infection in cells, and the TMPRSS2 inhibitor has been proposed as a potential treatment for COVID-19. A TMPRSS2 inhibitor has been highly efficient in preventing SARS-CoV-2 entry into cells.^[34]^ Although recombinant angiotensin-converting enzyme 2 has promising therapeutic potential against SARS-CoV,^[48]^ its fast clearance and short half-life in the circulation system limit its further application. A recombinant protein (ACE2-Ig), generated by fusing the extracellular domain of human ACE2 and Fc region of human IgG1, presents excellent pharmacological properties and high affinity to the receptor-binding domain of SARS-CoV and SARS-CoV-2 with promising outcome when used for diagnosis, prophylaxis, and treatment of SARS-CoV-2.^[49]^ The renin-angiotensin aldosterone system inhibitors are potential candidates for COVID-19 treatment (Clinical Trial NCT04311177); however, the balance between the circulating ACE2 and membrane-bound ACE2 receptor levels is crucial.^[50]^

ACE2 plays a vital role in maintaining endothelial integrity inside vessels.^[51]^ Vascular endothelial dysfunction can potentially initiate a coagulation cascade and eventually cause thrombosis. SARS-CoV binding can downregulate ACE2 expression, resulting in endothelial dysfunction.^[52]^ Interestingly, recombinant angiotensin-converting enzyme 2 blocks SARS-CoV-2 to infect engineered human vascular ECs.^[53]^

**Figure 2:**
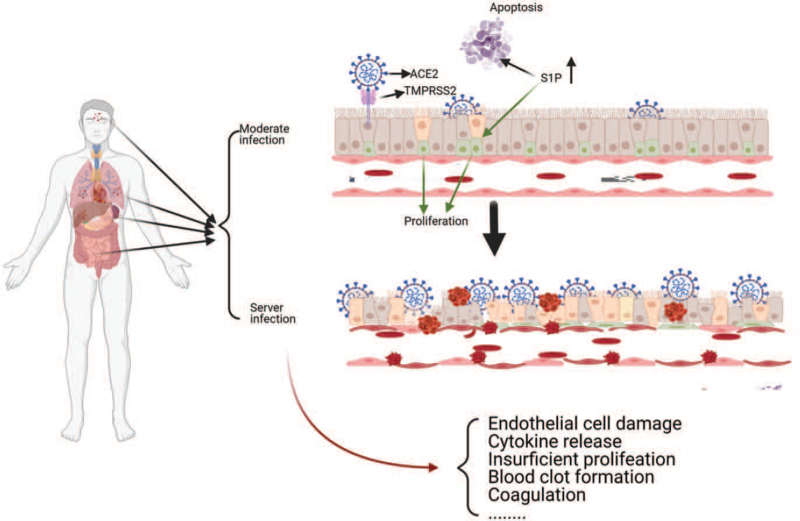
Different levels of severity of SARS-CoV-2 infection induce distinct endothelial cell damage in organs and tissues.

Biopsy of patients died by SARS-CoV-2 infection indicated that coronavirus particles commonly exist in the tubular epithelium and podocytes, except in renal ECs. Some segmental fibrin thrombi can be regarded as signs of severe endothelial injury in glomerular capillary loops. However, detailed mechanisms of endothelial injury and severity of illness by SARS-CoV-2 invasion remain unclear.^[54]^ Vascular injury is a prominent feature of severe SARS-CoV-2 infection that is commonly accompanied by cytoplasmic vacuolization and cell detachment in small and medium pulmonary arteries (Fig. 2), as observed in died individuals.^[55]^

Liver damage in SARS-CoV-infected patients has always been at the focus. Large numbers of virus particles widely exist in different organs, including the endothelial system,^[56,57]^ and SARS-CoV DNA has been detected in hepatocytes of affected individuals.^[58]^ Given that the entry of SARS-CoV into cells is facilitated by the ACE2 receptor,^[59]^ which is highly expressed in hepatic ECs,^[60]^ the liver is under high risk of SARS-CoV intrusion. In SARS-infected patients, significant pathological signs in mitotic cells suggest that SARS-CoV may induce apoptosis and cause liver injury. The SARS-CoV-specific protein 7a can induce apoptosis in different organs through the caspase-dependent pathway, suggesting the possibility of a direct attack on the liver by SARS-CoV.^[61]^

SARS-CoV-2 can attack ECs of the central nervous system through hematogenous and neuronal retrograde routes.^[62]^ In the neuronal retrograde route, neurotropic respiratory viruses reach neurons by retrograde axonal transportation, while viruses access neurons by infecting ECs across the hemato-encephalic barrier. A previous study has demonstrated that middle East respiratory syndrome coronavirus can enter the bloodstream after endothelial infection. The presence of viral particles in the brain capillary endothelium and active budding across ECs strongly suggest an endothelial pathway for SARS-CoV-2 infection in the brain.^[63]^

### 3.5 Therapeutic value of S1P to COVID-19

The primary targets of SARS-CoV-2 include nearly all vital organs and vascular ECs. In the lungs, alveolar damage and pulmonary microvascular thrombosis are major pathologies. Direct SARS-CoV-2 infection and the activation of other pathways eventually lead to endotheliopathy. As a result, vascular thrombotic events that occur in nearly all circulatory systems lead to multiorgan dysfunction and thrombotic complications. Additionally, vasculitis and systemic inflammatory vascular diseases are caused by endothelial damage resulting from SARS-CoV-2 infection. Therefore, we should focus on endotheliopathy, hypercoagulability, and vasculitis during clinical management of COVID-19 patients. Understanding the molecular mechanisms of infection and vascular damage by SARS-CoV-2, as well as the pathways involved in regulating endothelial dysfunction, may lead to the development of new therapeutic strategies against COVID-19, especially for the S1P application in this area.

## 4 Conclusion

Taken together, we conclude that maintaining or administering a high level of S1P will preserve the integrity of the EC structure and function, in turn, lowering the risk of SARS-CoV-2 infection and reducing complications and mortality.

## Author contributions

Jianshe Yang designed the study; Rongzhi Zhang, Qiang Wang, and Jianshe Yang wrote the manuscript. All authors have read and approved the final manuscript.

**Conceptualization:** Jianshe Yang.

**Data curation:** Rongzhi Zhang, Jianshe Yang.

**Formal analysis:** Qiang Wang.

**Funding acquisition:** Rongzhi Zhang, Qiang Wang, Jianshe Yang.

**Investigation:** Rongzhi Zhang, Qiang Wang, Jianshe Yang.

**Methodology:** Rongzhi Zhang, Jianshe Yang.

**Project administration:** Rongzhi Zhang, Qiang Wang.

**Resources:** Rongzhi Zhang, Qiang Wang, Jianshe Yang.

**Supervision:** Jianshe Yang.

**Validation:** Rongzhi Zhang, Qiang Wang, Jianshe Yang.

**Visualization:** Jianshe Yang.

**Writing – original draft:** Rongzhi Zhang, Qiang Wang, Jianshe Yang.

**Writing – review & editing:** Rongzhi Zhang, Jianshe Yang.
